# Supplementary material for: Heteroduplex oligonucleotide technology boosts oligonucleotide splice switching activity of morpholino oligomers in a Duchenne muscular dystrophy mouse model
Source: Nat Commun. 2024 Sep 26;15:7530. doi: 10.1038/s41467-024-48204-5 (PMC11427662; doi:10.1038/s41467-024-48204-5)
Supplement: Supplementary file 2 — Reporting Summary [file 41467_2024_48204_MOESM2_ESM.pdf]

nature portfolio

Corresponding author(s): Nagata T and Yokota T

Last updated by author(s): Feb 26, 2024

Reporting Summary

Nature Portfolio wishes to improve the reproducibility of the work that we publish. This form provides structure for consistency and transparency in reporting. For further information on Nature Portfolio policies, see our Editorial Policies and the Editorial Policy Checklist.

Statistics

For all statistical analyses, confirm that the following items are present in the figure legend, table legend, main text, or Methods section.

n/a	Confirmed	
☐	☒	The exact sample size (*n*) for each experimental group/condition, given as a discrete number and unit of measurement
☐	☒	A statement on whether measurements were taken from distinct samples or whether the same sample was measured repeatedly
☐	☒	The statistical test(s) used AND whether they are one- or two-sided<br>*Only common tests should be described solely by name; describe more complex techniques in the Methods section.*
☒	☐	A description of all covariates tested
☐	☒	A description of any assumptions or corrections, such as tests of normality and adjustment for multiple comparisons
☐	☒	A full description of the statistical parameters including central tendency (e.g. means) or other basic estimates (e.g. regression coefficient) AND variation (e.g. standard deviation) or associated estimates of uncertainty (e.g. confidence intervals)
☐	☒	For null hypothesis testing, the test statistic (e.g. *F*, *t*, *r*) with confidence intervals, effect sizes, degrees of freedom and *P* value noted<br>*Give P values as exact values whenever suitable.*
☒	☐	For Bayesian analysis, information on the choice of priors and Markov chain Monte Carlo settings
☒	☐	For hierarchical and complex designs, identification of the appropriate level for tests and full reporting of outcomes
☒	☐	Estimates of effect sizes (e.g. Cohen's *d*, Pearson's *r*), indicating how they were calculated

Our web collection on statistics for biologists contains articles on many of the points above.

Software and code

Policy information about availability of computer code

Data collection	Agilent- 2100 Expert Software
Data analysis	Agilent-2100 Expert Software<br>Excel for Microsoft 365 MSO(16.0.16026.20002)<br>Graphpad prism 9.4.0<br>HALO Image Analysis<br>Imaris ver. 9.7.0

For manuscripts utilizing custom algorithms or software that are central to the research but not yet described in published literature, software must be made available to editors and reviewers. We strongly encourage code deposition in a community repository (e.g. GitHub). See the Nature Portfolio guidelines for submitting code & software for further information.

Data

Policy information about availability of data

All manuscripts must include a data availability statement. This statement should provide the following information, where applicable:

- Accession codes, unique identifiers, or web links for publicly available datasets
- A description of any restrictions on data availability
- For clinical datasets or third party data, please ensure that the statement adheres to our policy

All data supporting the findings of this study are available within the paper and supplementary information files. Source data are provided with this paper.

Human research participants

Policy information about studies involving human research participants and Sex and Gender in Research.

Reporting on sex and gender	Not applicable
Population characteristics	Not applicable
Recruitment	Not applicable
Ethics oversight	Not applicable

Note that full information on the approval of the study protocol must also be provided in the manuscript.

Field-specific reporting

Please select the one below that is the best fit for your research. If you are not sure, read the appropriate sections before making your selection.

☒ Life sciences ☐ Behavioural & social sciences ☐ Ecological, evolutionary & environmental sciences

For a reference copy of the document with all sections, see nature.com/documents/nr-reporting-summary-flat.pdf

Life sciences study design

All studies must disclose on these points even when the disclosure is negative.

Sample size	No sample size calculations were performed beforehand. Sample size was determined to be adequate based on previously published researches. In all experiments, more than 4 mice per group were used for analysis.
Data exclusions	No samples were excluded from the analysis.
Replication	Experiments were repeated with similar results at least two times with independent biological replicates. Number of reliable reproductions of each experimental finding is indicated in each figure legend.
Randomization	Allocation of animals to the different groups was random by trained researchers performing each experiment.
Blinding	Blinding was not used to prevent sample misidentification. Bias cannot change the results of delivery efficiency and skipping patterns.

Reporting for specific materials, systems and methods

We require information from authors about some types of materials, experimental systems and methods used in many studies. Here, indicate whether each material, system or method listed is relevant to your study. If you are not sure if a list item applies to your research, read the appropriate section before selecting a response.

Materials & experimental systems

n/a	Involved in the study	
☐	☒	Antibodies
☒	☐	Eukaryotic cell lines
☒	☐	Palaeontology and archaeology
☐	☒	Animals and other organisms
☒	☐	Clinical data
☒	☐	Dual use research of concern

Methods

n/a	Involved in the study	
☒	☐	ChIP-seq
☒	☐	Flow cytometry
☒	☐	MRI-based neuroimaging

Antibodies

Antibodies used

anti-dystrophin antibody (ab15277, 1:200; Abcam, Cambridge, UK)
anti-vinculin antibody (NB600-1293, 1:10000; Novus Biologicals, Centennial, CO, USA)
mouse anti-α-sarcoglycan (NCL-L-a-SARC, 1:200; Leica Biosystems)
mouse anti-β-dystroglycan (NCL-b-DG, 1:200; Leica Biosystems)
anti-nNOS (#61-7000-rabbit, 1:1000; Thermo Fisher Scientific)
mouse anti-Caveolin 3 (sc-5310, 1:500; Santa Cruz Biotechnology).
mouse anti-lamin A/C antibody (8617s, 1:100; Cell Signal Technology, Alexa Fluor488 Conjugate)
anti-wheat germ agglutinin lectin (W11262, 1:300; Thermo Fisher Scientific Inc., Alexa Fluor594 Conjugate)
Goat anti-Mouse IgG (H+L) Highly Cross-Adsorbed Secondary Antibody, Alexa Fluor™ 546 (A-11030, 1:1000; Thermo Ficher Scientific)
Goat anti-Rabbit IgG (H+L) Cross-Adsorbed Secondary Antibody, Alexa Fluor™ 568 (A-11011, 1:1000; Thermo Ficher Scientific)
Peroxidase AffiniPure™ Goat Anti-Rabbit IgG (H+L) (#111-035-003, 1:3000; Jackson ImmunoResearch)
Peroxidase AffiniPure™ Goat Anti-Mouse IgG (H+L) (#115-035-003, 1:10,000; Jackson ImmunoResearch)

Validation

All the antibodies used in the study were validated by the manufacture.
anti-dystrophin: https://www.abcam.co.jp/products/primary-antibodies/dystrophin-antibody-ab15277.html
anti-vinculin: https://www.novusbio.com/products/vinculin-antibody-hvin-1_nb600-1293
anti-α-sarcoglycan: https://shop.leicabiosystems.com/ja-jp/ihc-ish/ihc-primary-antibodies/pid-A-SARC-L-CE
anti-β-dystroglycan: https://shop.leicabiosystems.com/ja-jp/ihc-ish/ihc-primary-antibodies/pid-B-DG-CE
anti-nNOS: https://www.thermofisher.com/order/genome-database/generatePdf?productName=nNOS&assayType=PRANT&productId=61-7000&detailed=true
anti-caveolin 3: https://datasheets.scbt.com/sc-5310.pdf
anti-lamin A/C: https://www.cellsignal.jp/products/antibody-conjugates/lamin-a-c-4c11-mouse-mab-alexa-fluor-488-conjugate/8617
anti-wheat germ agglutinin lectin: https://www.thermofisher.com/order/catalog/product/jp/ja/W11262
anti-Mouse IgG with Alexa 546: https://www.thermofisher.com/antibody/product/Goat-anti-Mouse-IgG-H-L-Highly-Cross-Adsorbed-Secondary-Antibody-Polyclonal/A-11030
anti-Rabbit IgG with Alexa 568: https://www.thermofisher.com/antibody/product/Goat-anti-Rabbit-IgG-H-L-Cross-Adsorbed-Secondary-Antibody-Polyclonal/A-11011
anti-Rabbit IgG with Peroxidase: https://www.jacksonimmuno.com/catalog/products/111-035-003
anti-Mouse IgG with Peroxidase: https://www.jacksonimmuno.com/catalog/products/115-035-003

Animals and other research organisms

Policy information about studies involving animals; ARRIVE guidelines recommended for reporting animal research, and Sex and Gender in Research

Laboratory animals

Mice (mdx, C57BL/10ScSn-Dmdmdx/J, 6-8-week-old males, and C57BL/10ScNJic [B10], 6-8-week-old males) were purchased from the CLEA Japan Laboratories. Animals were housed under specific pathogen-free conditions (temperature: 18–24°C; humidity: 40–70%) in a day-night controlled light cycle, provided with food and water ad libitum.

Wild animals

Study did not involve wild animals.

Reporting on sex

Since DMD is the most common muscular dystrophy in children affecting primarily boys, the male mdx mouse was used in this experiment.

Field-collected samples

Study did not involve samples collected from the field.

Ethics oversight

The experimental procedures used in this study were approved by the Institutional Animal Care and Use Committee of Tokyo Medical and Dental University (Approval number A2022-085A).

Note that full information on the approval of the study protocol must also be provided in the manuscript.
