# Supplementary material for: Heteroduplex oligonucleotide technology boosts oligonucleotide splice switching activity of morpholino oligomers in a Duchenne muscular dystrophy mouse model
Source: Nat Commun. 2024 Sep 26;15:7530. doi: 10.1038/s41467-024-48204-5 (PMC11427662; doi:10.1038/s41467-024-48204-5)
Supplement: Supplementary file 3 — Supplementary information [file 41467_2024_48204_MOESM3_ESM.pdf]

Supplementary information

Title: Heteroduplex oligonucleotide technology boosts oligonucleotide splice switching activity of morpholino oligomers in a Duchenne muscular dystrophy mouse model

Authors: Juri Hasegawa[1, 2, 9], Tetsuya Nagata[1, 2, 3, 9, *], Kensuke Ihara[4, 5], Jun Tanihata[6], Satoe Ebihara[1, 2], Kie Yoshida-Tanaka[1, 2], Mitsugu Yanagidaira[1, 2], Masahiro Ohara[1, 2], Asuka Sasaki[1, 2], Miyu Nakayama[7], Syunsuke Yamamoto[7], Takashi Ishii[1, 2], Rintaro Iwata-Hara[1, 2], Mitsuru Naito[8], Kanjiro Miyata[8], Fumika Sakaue[1, 2] & Takanori Yokota[1, 2, 3, *]

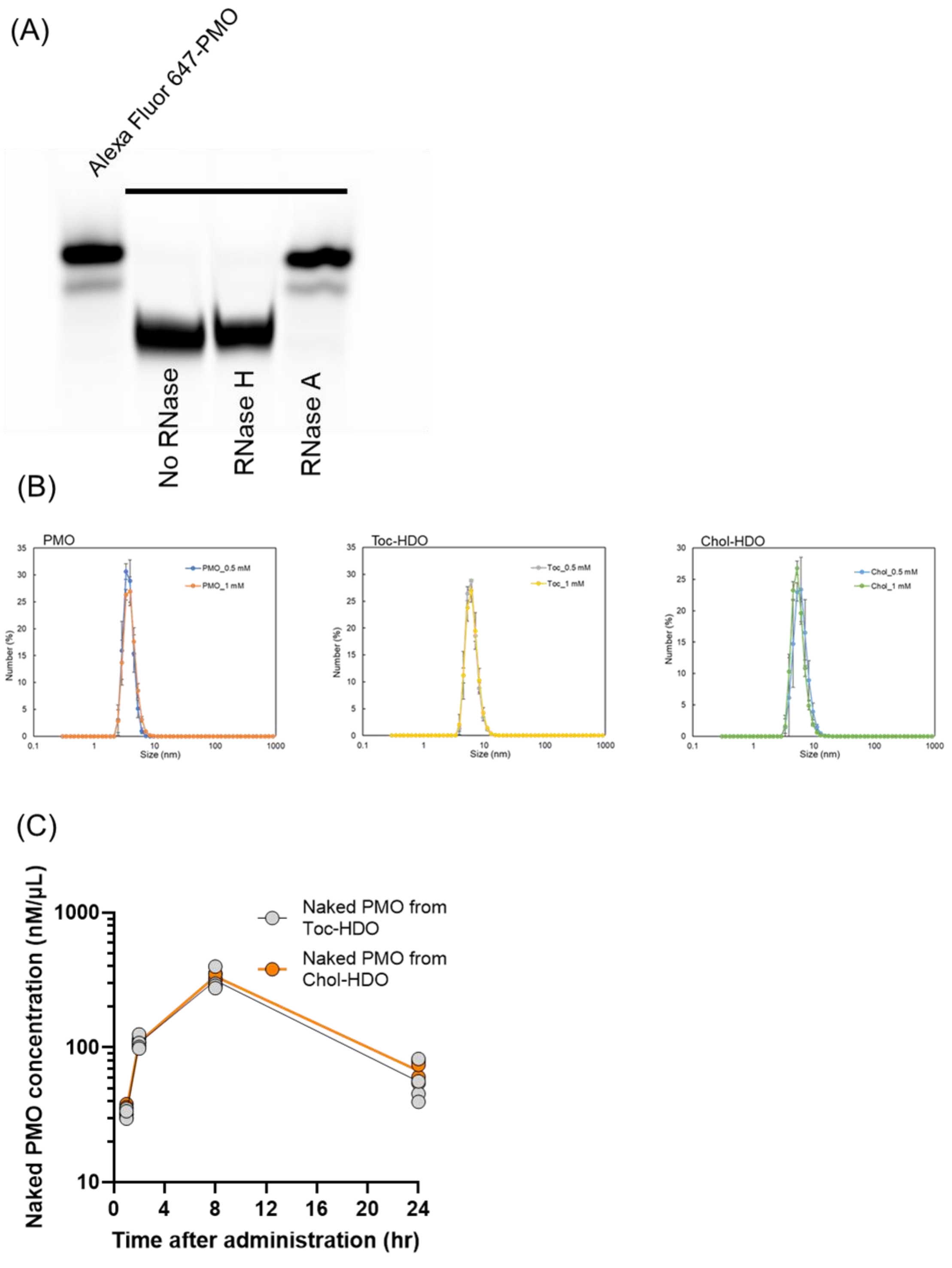

(A)
Alexa Fluor 647-PMO
No RNase
RNase H
RNase A
(B)
PMO
PMO_0.5 mM
PMO_1 mM
Toc-HDO
Toc_0.5 mM
Toc_1 mM
Chol-HDO
Chol_0.5 mM
Chol_1 mM
Number (%)
Size (nm)
(C)
Naked PMO concentration (nM/μL)
Time after administration (hr)
Naked PMO from Toc-HDO
Naked PMO from Chol-HDO

(D) PMO detection using ISH (RNA scope) Heart

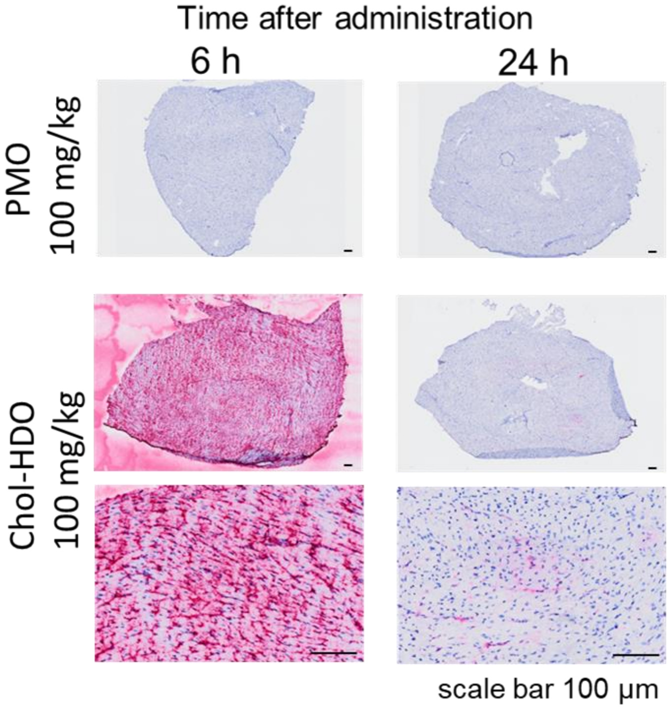

(E) PMO detection using ISH (RNA scope) Quadriceps

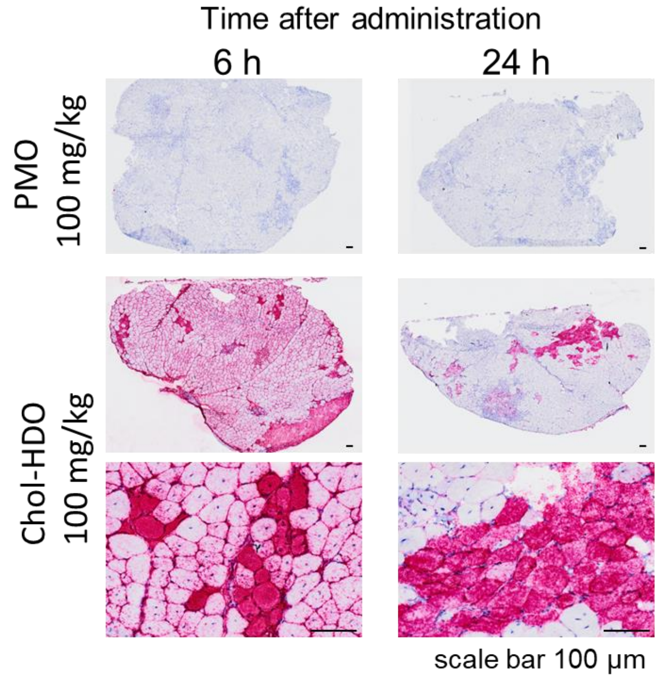

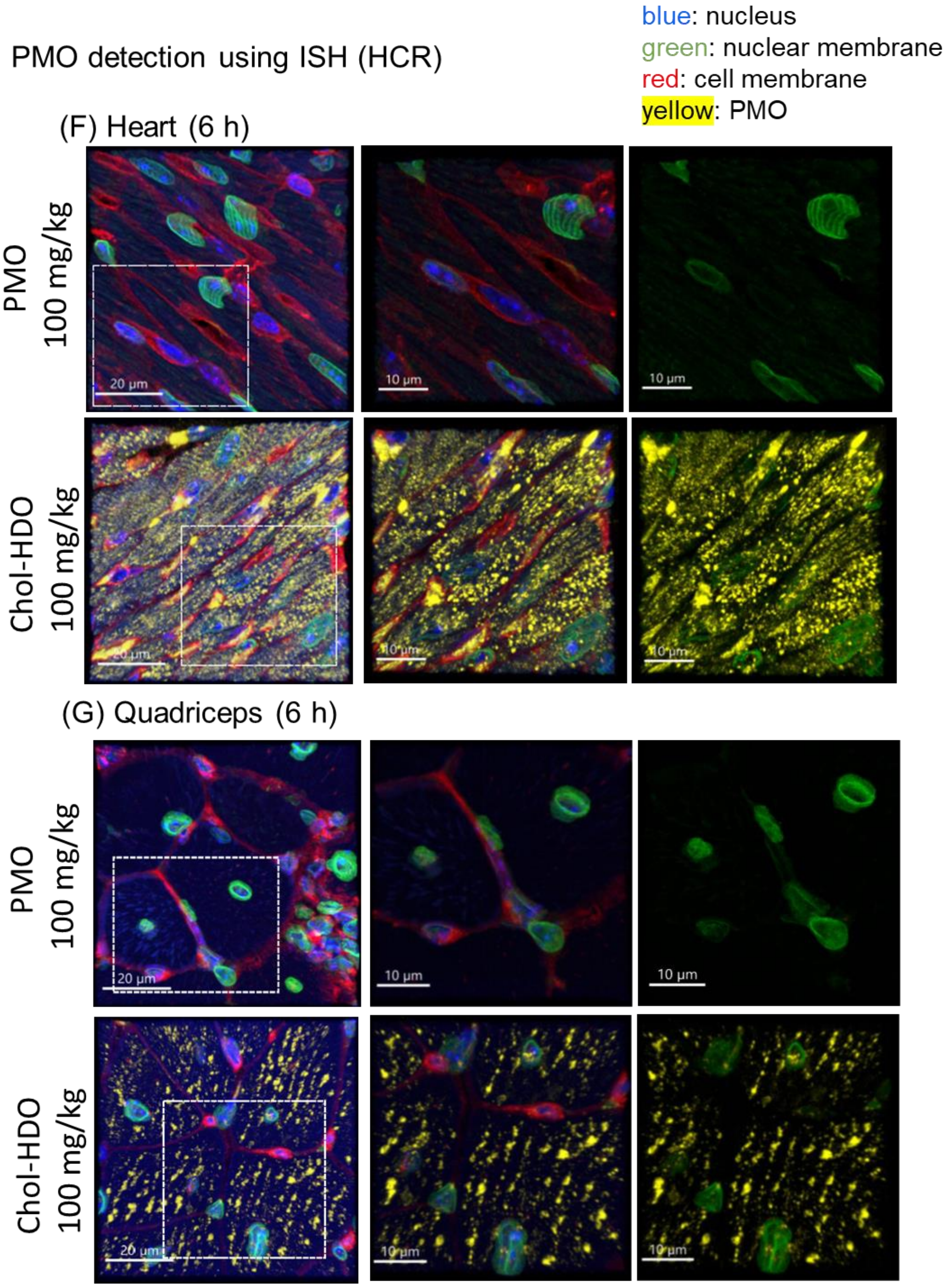

Supplementary Fig. 1 Pharmacokinetics of PMO and Chol-HDO in the heart and QC

(A) Sensitivity of phosphorodiamidate morpholino oligomer（PMO) /HDO to RNase A and H. (B) The histogram of PMO and PMO/HDOs were measured by dynamic light

scattering. The histogram showed a narrow size distribution of less than 10 nm in diameter, suggesting that no aggregates were formed. (C) Pharmacokinetics of naked PMO from tocopherol（Toc)-HDO (PMO) and cholesterol（Cho)-HDO (PMO) after intravenous injection of a single Toc-HDO or Chol-HDO dose (11.88 μmol/kg). The hybridization-based ELISA shows the pharmacokinetics of Toc-HDO or Chol-HDO treated without *E.coli*-derived RNase A in *mdx* mice. (n = 4 per group) (D) Time course of PMO distribution detected via *in situ* hybridization using the probe complementary to the PMOs in the heart at 6 and 24 h, after a single intravenous injection of 100 mg/kg (11.88 μmol/kg) of PMO or the same molar equivalent of Chol-HDO. Scale bar, 100 μm. (E) Time course of PMO distribution detected via *in situ* hybridization in the QF at 6 and 24 h after a single intravenous injection of 100 mg/kg (11.88 μmol/kg) of PMO or same molar equivalent of Chol-HDO. Scale bar, 100 μm. (F) PMO distribution (Cy5 (yellow)) detected via *in situ* hybridization chain reaction (HCR) using the probe complementary to the PMOs with staining by anti-lamin A/C (Alexa488 (red)), anti-wheat germ agglutinin lectin (Alexa594 (green)) and DAPI (blue) in the heart at 6 h, after a single intravenous injection of 100 mg/kg (11.88 μmol/kg) of PMO or the same molar equivalent of Chol-HDO. Scale bar, 20 μm (left panel). Scale bar, 10 μm (middle and right panel). (G) PMO distribution detected via in situ HCR in the quadriceps at 6 h, after a single intravenous injection of 100 mg/kg of PMO or the same molar equivalent of Chol-HDO (11.88 μmol/kg). Scale bar, 20 μm (left panel). Scale bar, 10 μm (middle and right panel). Source data are provided as a Source Data file.

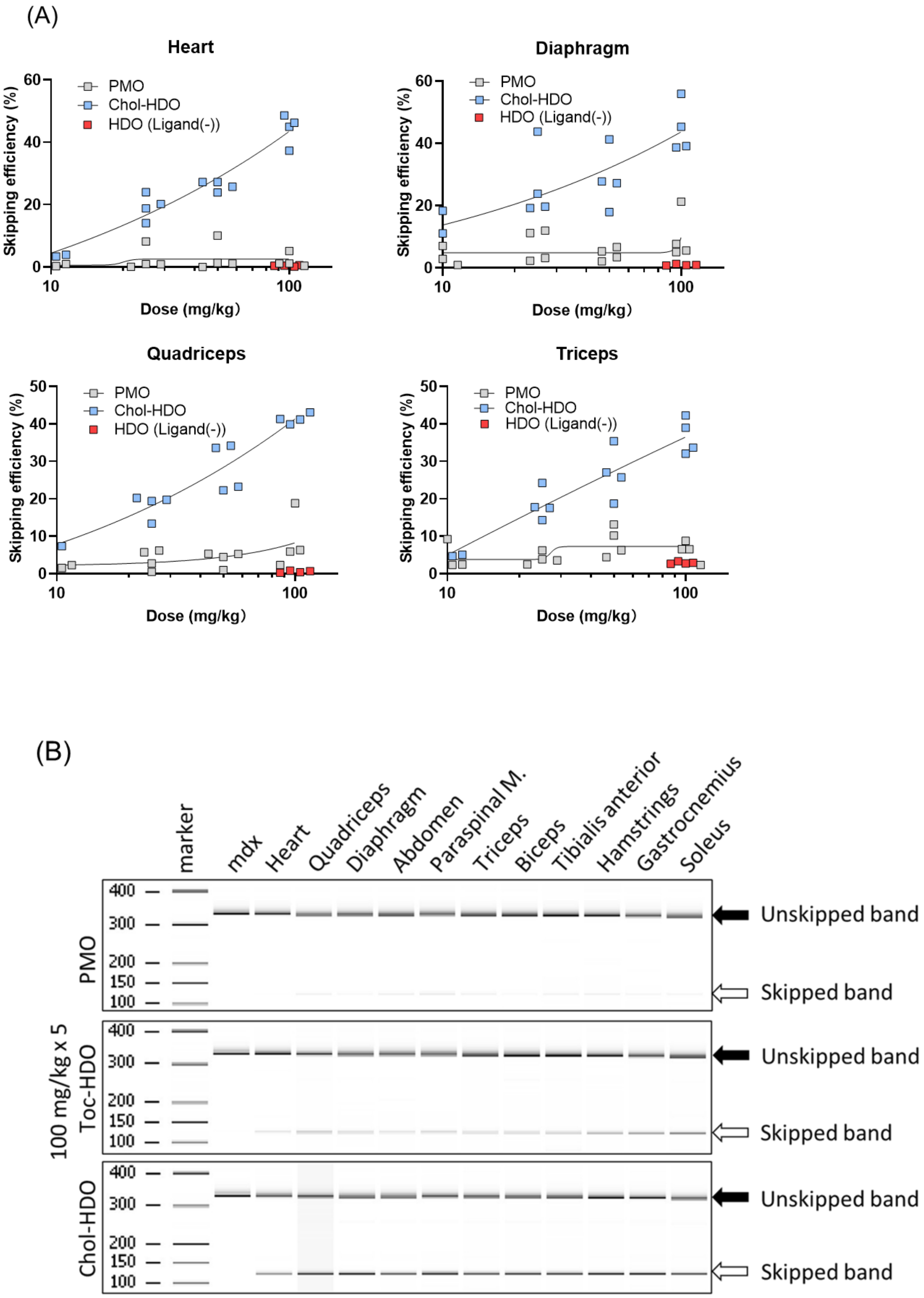
(A)
Heart
Diaphragm
Quadriceps
Triceps
PMO
Chol-HDO
HDO (Ligand(-))
Skipping efficiency (%)
Dose (mg/kg)
(B)
marker
mdx
Heart
Quadriceps
Diaphragm
Abdomen
Paraspinal M.
Triceps
Biceps
Tibialis anterior
Hamstrings
Gastrocnemius
Soleus
PMO
Toc-HDO
Chol-HDO
100 mg/kg x 5
Unskipped band
Skipped band

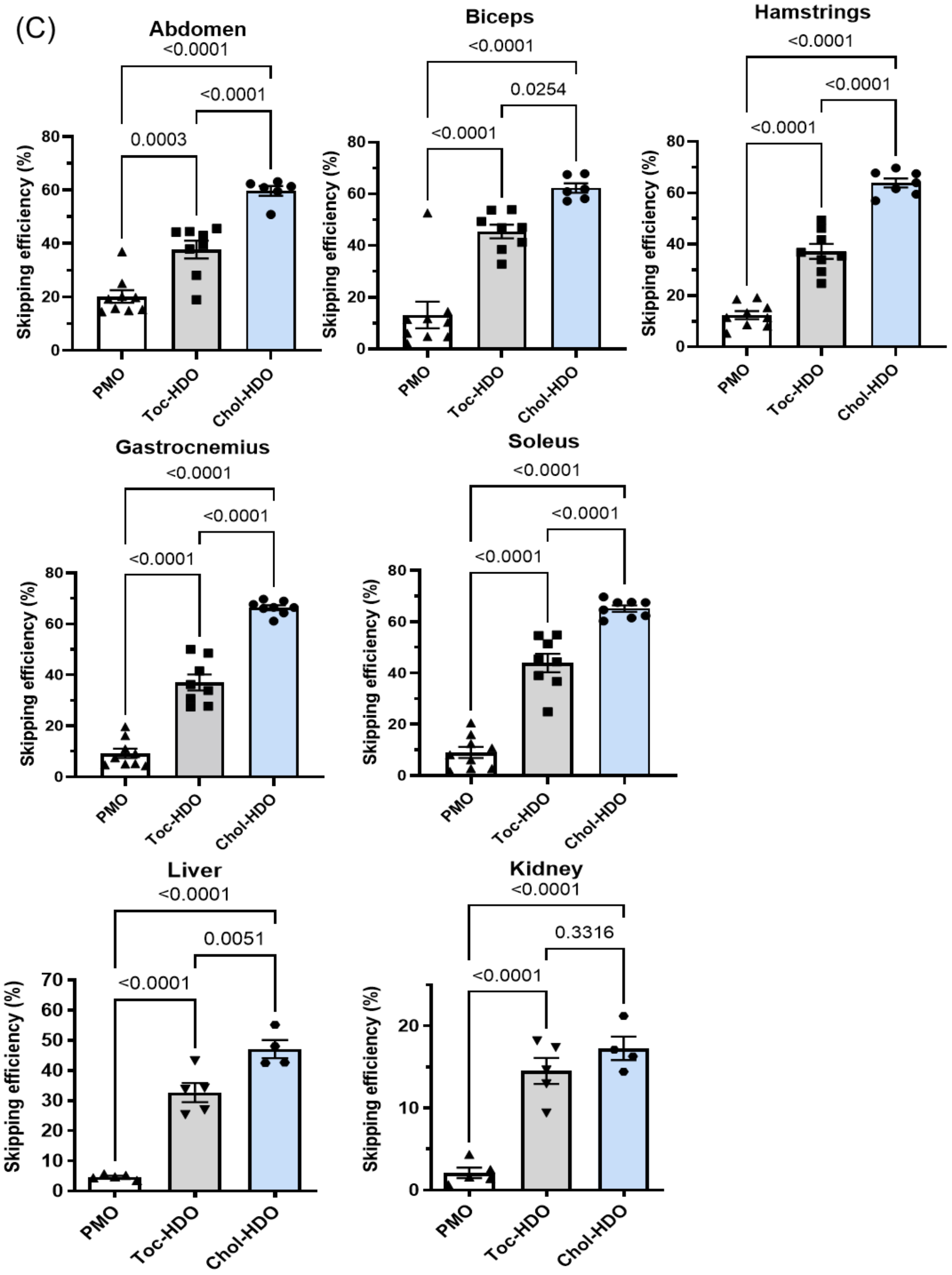

Supplementary Fig. 2 Comparison of the effects of PMO and PMO/HDO on the efficiency of exon 23 skipping.

(A) Dose–response curve showing the skipping efficiency for the heart, diaphragm,

quadriceps (QF), or triceps after a single intravenous (IV) injection of PMO or Chol-HDO (dose for PMO, 10, 25, 50, or 100 mg/kg; dose for Chol-HDO, same equimolar doses of PMO; n = 4 per group). Muscles were collected 2 weeks after injection. The red squares represent the skipping efficiency when PMO/HDO without any ligand was administered at a molar equivalent of 100 mg/kg of PMO (11.88 μmol/kg). (B) Representative images of exon 23 skipping analyzed using the bioanalyzer 2100 in indicated tissues 2 weeks after five weekly injections of PMO, Toc-, or Chol-HDO (11.88 μmol/kg). (C) Detection of exon 23-skipped dystrophin mRNA in the indicated muscles, liver, or kidney 2 weeks after five weekly injections of PMO, Toc-, or Chol-HDO (11.88 μmol/kg) (n = 4-9 per group). Data are presented as mean ± S.E.M. and were analyzed using one-way analysis of variance followed by Tukey's Kramer tests. P values are indicated. Source data are provided as a Source Data file.

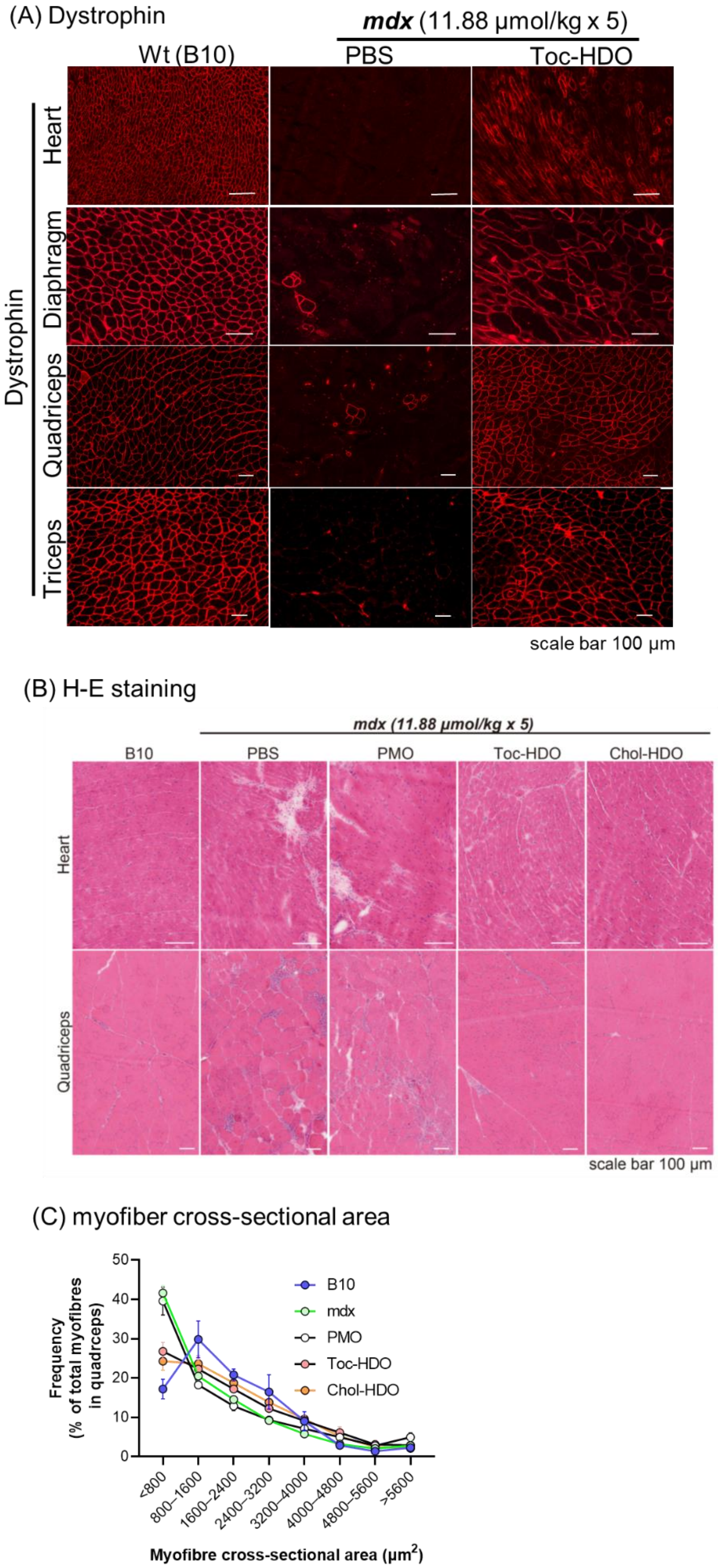
(A) Dystrophin
mdx (11.88 µmol/kg x 5)
Wt (B10)
PBS
Toc-HDO
Dystrophin
Heart
Diaphragm
Quadriceps
Triceps
scale bar 100 µm
(B) H-E staining
mdx (11.88 µmol/kg x 5)
B10
PBS
PMO
Toc-HDO
Chol-HDO
Heart
Quadriceps
scale bar 100 µm
(C) myofiber cross-sectional area
Frequency (% of total myofibres in quadrceps)
B10
mdx
PMO
Toc-HDO
Chol-HDO
<800
800–1600
1600–2400
2400–3200
3200–4000
4000–4800
4800–5600
>5600
Myofibre cross-sectional area (µm2)

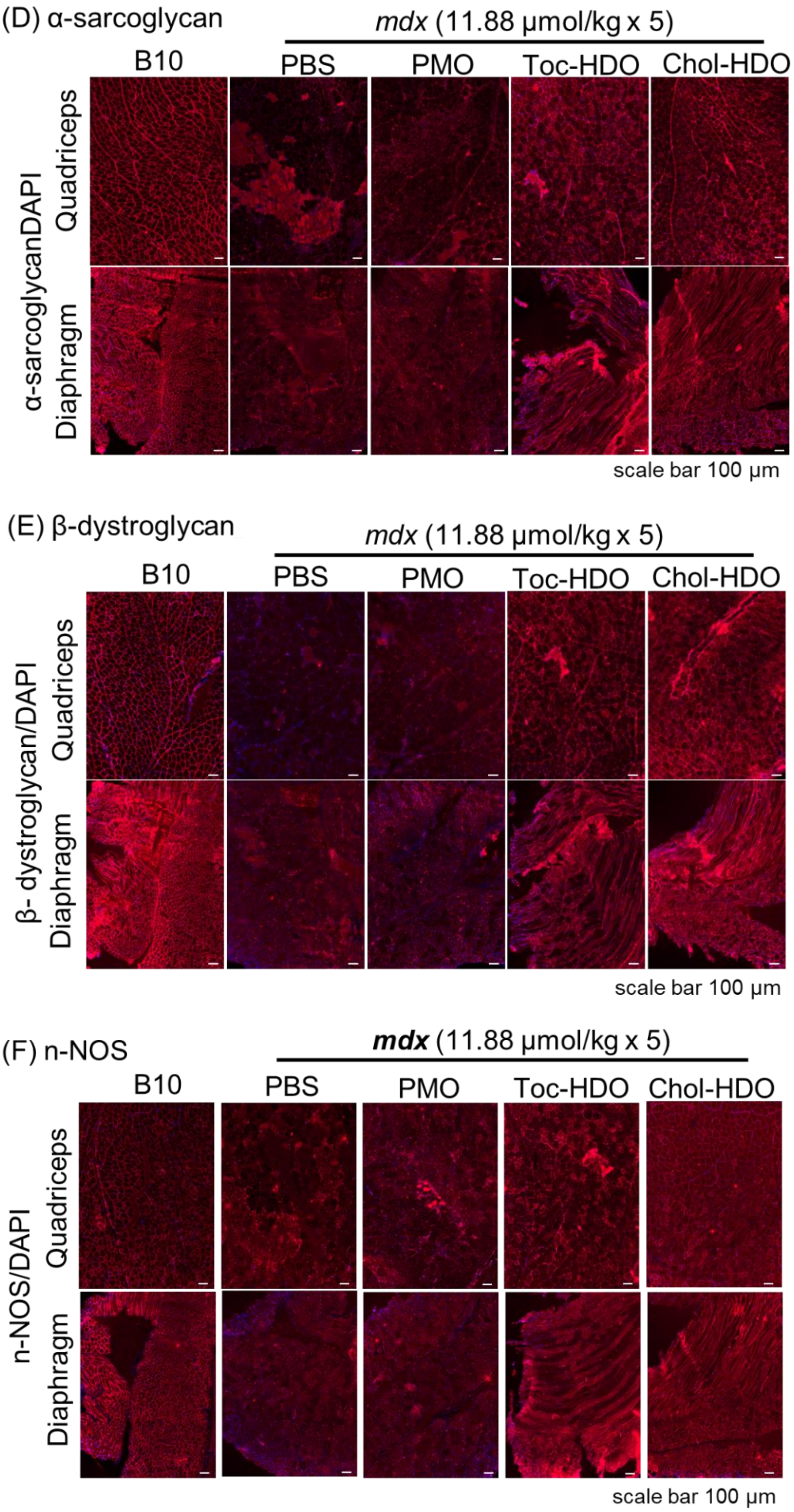
(D) α-sarcoglycan
mdx (11.88 μmol/kg x 5)
B10
PBS
PMO
Toc-HDO
Chol-HDO
α-sarcoglycanDAPI
Quadriceps
Diaphragm
scale bar 100 μm
(E) β-dystroglycan
mdx (11.88 μmol/kg x 5)
B10
PBS
PMO
Toc-HDO
Chol-HDO
β- dystroglycan/DAPI
Quadriceps
Diaphragm
scale bar 100 μm
(F) n-NOS
mdx (11.88 μmol/kg x 5)
B10
PBS
PMO
Toc-HDO
Chol-HDO
n-NOS/DAPI
Quadriceps
Diaphragm
scale bar 100 μm

Supplementary Fig. 3 Immunofluorescence analysis of dystrophin and dystrophin-associated proteins.

(A) Representative images of dystrophin immunostaining in the indicated muscle tissues 2 weeks after five weekly injections of Toc-HDO (11.88 μmol/kg) compared to that in B10 or phosphate-buffered saline (PBS)-treated *mdx*. Scale bar, 100 μm. (B) Hematoxylin and eosin staining of the heart and QF 2 weeks after five weekly injections of PMO, Toc-, or Chol-HDO (11.88 μmol/kg) compared to that in B10 or PBS-treated *mdx*. (C) Myofiber cross-sectional area of the QF 2 weeks after five weekly injections of PMO, Toc-, or Chol-HDO (11.88 μmol/kg) compared to that in B10 or PBS-treated *mdx* (n = 4 per group). Data are presented as mean ± S.E.M. (D/E/F) Immunofluorescence analysis of dystrophin-associated proteins 2 weeks after five weekly injections of PMO, Toc-, or Chol-HDO (11.88 μmol/kg) compared to that in B10 or PBS-treated *mdx*. Representative images from control and treated QF and diaphragm muscles stained with α-sarcoglycan, β-dystroglycan, and neuronal nitric oxide synthase. Scale bar, 100 μm. Source data are provided as a Source Data file.

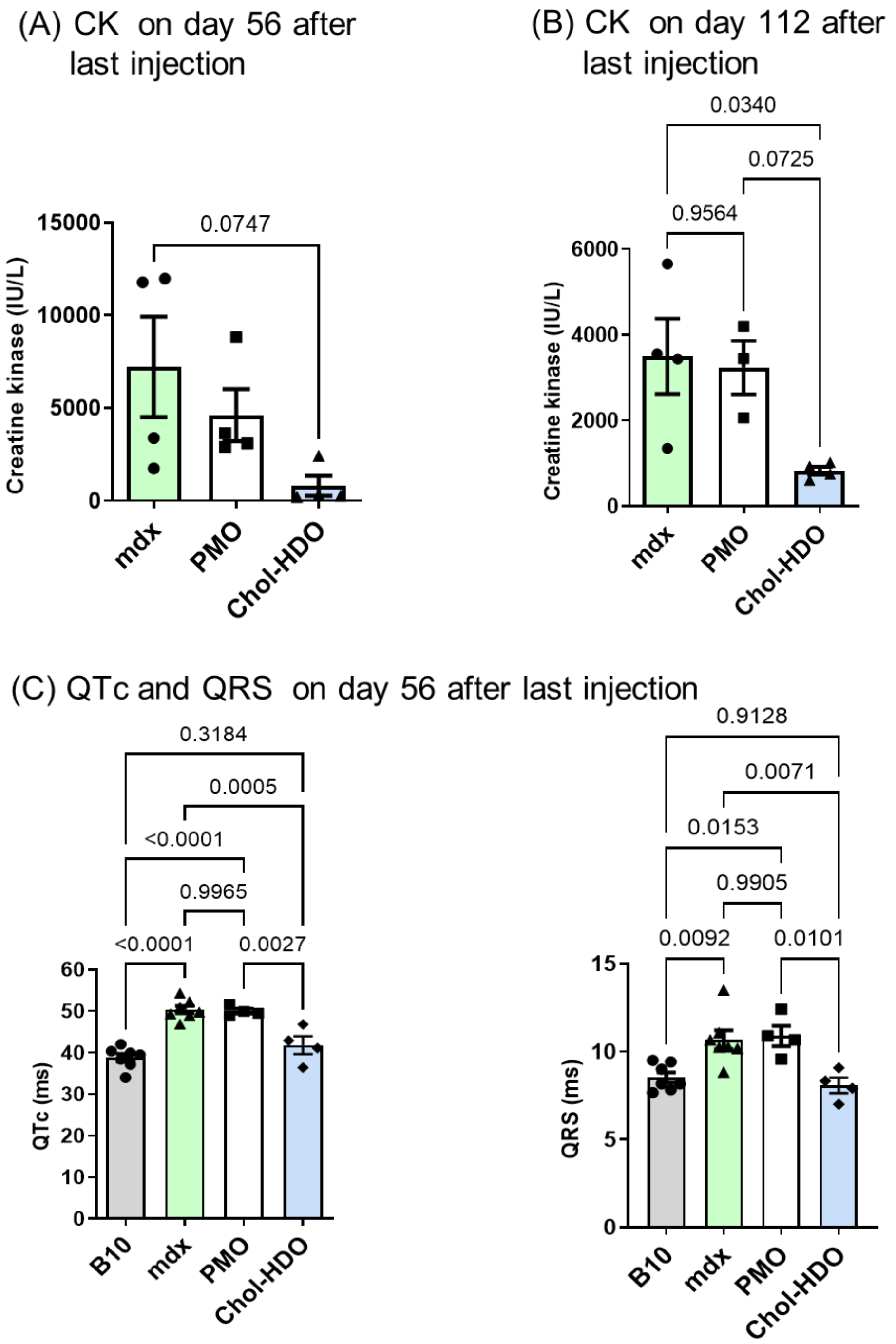

Supplementary Fig. 4 Serum biomarkers levels and ECG change in *mdx* treated mice.

(A) Serum Creatine Kinase (CK) levels 56 days after five weekly systemic injections of PMO or Chol-HDO (11.88 μmol/kg) compared to those in PBS-treated *mdx* (n = 4 per

group). (B) Serum CK levels 112 days after the last of five weekly systemic injections of PMO or Chol-HDO (11.88 μmol/kg) compared to those in PBS-treated *mdx* (n = 3-4 per group). (C) Electrocardiography (ECG) abnormalities (QTc and QRS duration) observed in *mdx* were still prevented 56 days after five weekly systemic injections of PMO or Chol-HDO (11.88 μmol/kg) compared to the abnormalities in PBS-treated *mdx* (n = 4-7 per group).

Data are presented as mean ± S.E.M. and were analyzed using one-way analysis of variance followed by Tukey's tests or Tukey's Kramer tests. P values are indicated. Source data are provided as a Source Data file.

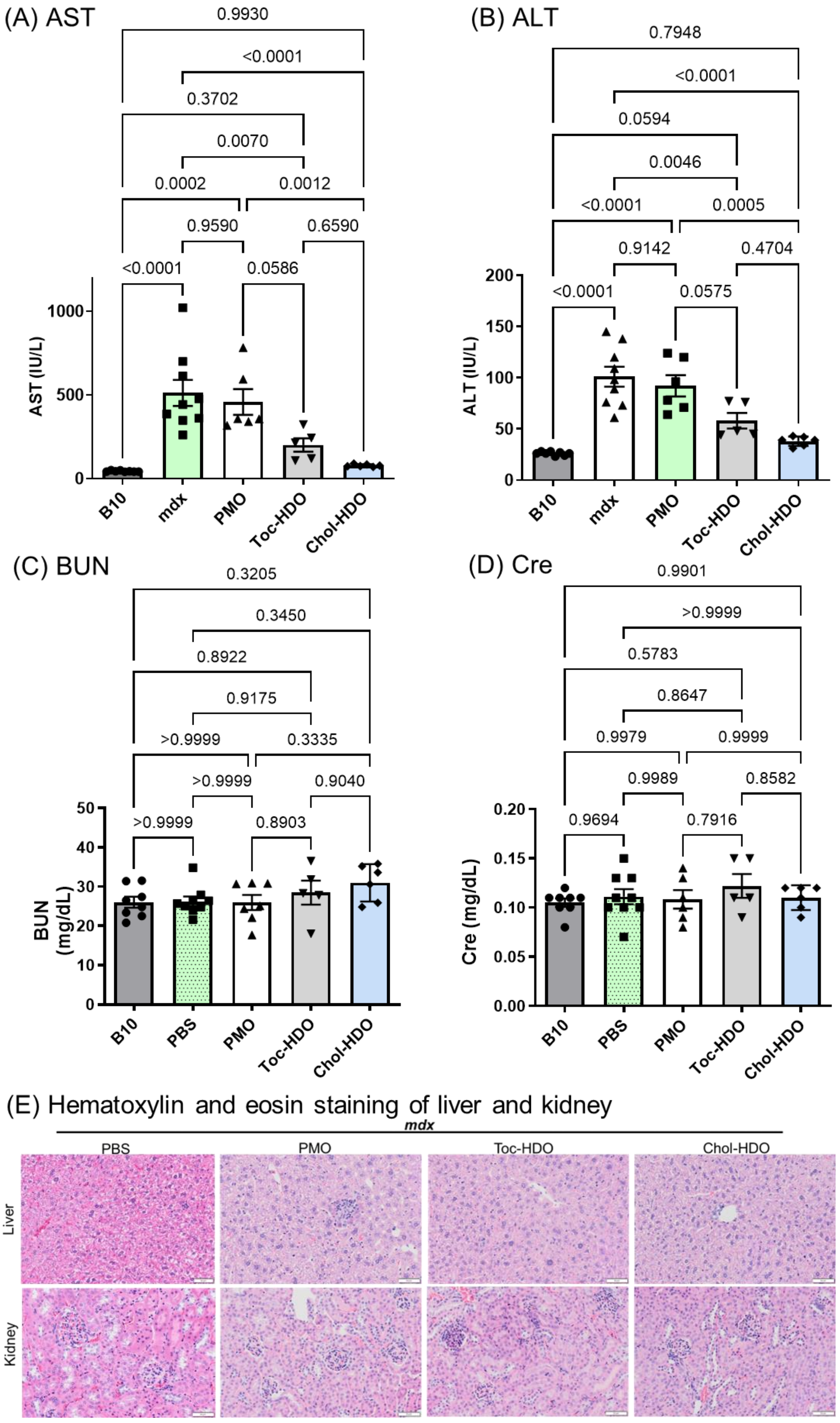
(A) AST
0.9930
<0.0001
0.3702
0.0070
0.0002
0.0012
0.9590
0.6590
<0.0001
0.0586
AST (IU/L)
1000
500
0
B10
mdx
PMO
Toc-HDO
Chol-HDO
(B) ALT
0.7948
<0.0001
0.0594
0.0046
<0.0001
0.0005
0.9142
0.4704
<0.0001
0.0575
ALT (IU/L)
200
150
100
50
0
B10
mdx
PMO
Toc-HDO
Chol-HDO
(C) BUN
0.3205
0.3450
0.8922
0.9175
>0.9999
0.3335
>0.9999
0.9040
>0.9999
0.8903
BUN (mg/dL)
50
40
30
20
10
0
B10
PBS
PMO
Toc-HDO
Chol-HDO
(D) Cre
0.9901
>0.9999
0.5783
0.8647
0.9979
0.9999
0.9989
0.8582
0.9694
0.7916
Cre (mg/dL)
0.20
0.15
0.10
0.05
0.00
B10
PBS
PMO
Toc-HDO
Chol-HDO
(E) Hematoxylin and eosin staining of liver and kidney
mdx
PBS
PMO
Toc-HDO
Chol-HDO
Liver
Kidney

Supplementary Fig. 5 Evaluation of liver and kidney function and histopathology following multiple administrations of PMO, Toc-HDO, or Chol-HDO

Serum levels of aspartate aminotransferase (AST) (A), alanine aminotransferase (ALT) (B), blood urea nitrogen (BUN) (C), and creatinine (Cre) (D), were measured in mice treated with PMO, Toc-, or Chol-HDO 2 weeks after five weekly systemic injections (n = 5-9 per group). Data are presented as mean ± S.E.M. and were analyzed using one-way analysis of variance followed by Tukey's Kramer tests. P values are indicated. (E) Histological analysis using hematoxylin and eosin staining of *mdx* mice treated with PBS, PMO, Toc-HDO or Chol-HDO. In liver (upper panel), small foci of inflammatory cell infiltration were scattered in the liver parenchyma of *mdx* mice treated with PBS. No additional lesions were found in *mdx* mice with Chol-HDO. In *mdx* mice treated with Toc-HDO, an increase in size heterogeneity of hepatocyte nuclei was sometimes noted, occasionally with meganucleation. In kidney (lower panel), no lesions were observed in *mdx* mice following treatment with PBS or Chol-HDO. A slight increase in cellular density in glomeruli was sometimes noted in *mdx* mice treated with Toc-HDO. Scale bar, 50 μm Source data are provided as a Source Data file.

Supplementary table. 1. HDO melting temperatures

	Melting temperature (℃)
Toc-HDO (PMO/RNA gap)	78.39
Chol-HDO (PMO/RNA gap)	84.48
Chol-HDO (PMO/DNA gap)	73.744
Chol-HDO (PMO/OMe gap)	86.7
